# Development and Improvement of Methods to Disinfect Raw Beef Using Calcium Hydroxide–Ethanol–Lactate-Based Food Disinfectant for Safe Consumption

**DOI:** 10.3389/fmicb.2020.537889

**Published:** 2020-11-20

**Authors:** Ahmad Yaman Kayali, Jo Ozawa, Mitsuaki Nishibuchi

**Affiliations:** ^1^Division of Environmental Coexistence, Center for Southeast Asian Studies, Kyoto University, Kyoto, Japan; ^2^T. K. Shin Co., Ltd., Hyogo, Japan

**Keywords:** raw beef, disinfection, method, enterohemorrhagic *Escherichia coli*, *Salmonella*, calcium hydroxide–ethanol–lactate (CEL) disinfectant, detection, loop-mediated isothermal amplification

## Abstract

The enterohemorrhagic *Escherichia coli* (EHEC) group is responsible for outbreaks and sporadic cases around the world annually. EHEC produces a potent protein known as Shiga toxin in the human intestine causing mild to bloody diarrhea. Some cases of EHEC infections may develop life-threatening symptoms, which may lead to human death. It also has other virulent factors that enable the EHEC cells to adhere to a target tissue and invade to some extent to crave more nutrition and escape the external extreme conditions, such as disinfection treatment. For those reasons, beef is not permitted for raw consumption unless guaranteed free of harmful bacteria, including EHEC, or the invading bacterial cells are completely removed or reduced to a safe level. A heat treatment that guarantees a sufficiently high temperature to reach inside the tissue of meat through the surface was established in Japan. This treatment may allow the core part of the meat to be consumed raw. However, it seemed to have some limitations. We aimed at developing a disinfection method with, hypothetically, nutrition-preserving property that is equivalent to the heat treatment or even superior. A combination of calcium hydroxide–ethanol–lactate-based food disinfectant and two proposed physical sterilization methods, assisted with microbial detection methods, exerted sufficient bactericidal activities against EHEC cells adhering to and/or invading the beef. These physical methods showed great usefulness in disinfecting fresh full-size boneless Round-beef of around 12 kg including fat on the outside. The first method applied a commercially available wide-drum washing machine (WM method) while the second method applied a specially designed plastic bag and a commercially available vibration machine (VV method). After trimming out the fat and the denatured surface of the beef (1 cm from the surface), the remaining meat mass showed no signs of denaturation and a significant reduction of viable EHEC cells by a factor of >10^4^ CFU/ml. However, in the WM method, the disinfection process required a large amount of the disinfectant (150 L). The improved method, VV method, implemented a system that consumes a smaller amount of the disinfectant (50 L) while ensuring the targeted disinfection power degree.

## Introduction

Enterohemorrhagic *Escherichia coli* (EHEC) are a group of pathogenic *E. coli*. It is prevalent in cattle’s intestine being a natural reservoir of EHEC. It contaminates human’s food and water directly or indirectly, causing outbreaks and a large number of sporadic cases around the world. Unlike the non-pathogenic *E. coli* strains, EHEC produces the Shiga toxin that harms the internal surface of the intestine, resulting in mild to bloody diarrhea in humans. Some cases of EHEC infections may develop hemolytic uremic syndrome (HUS) that may lead to human death (Jubelin et al., 2018; Rojas-Lopez et al., 2018). EHEC is considered an important cause of illness and death among all the other foodborne pathogens in Japan, the United States of America, and others. Incidentally, 30,871 cases of EHEC were reported between 2011 and 2018 in Japan, including 61 fatal cases, according to the National Institute of Infectious Diseases, Ministry of Health, Labour and Welfare of Japan (Infectious Agents Surveillance Report [IASR], 2012, 2013, 2014, 2015, 2016, 2017, 2018, 2019). Meats might be contaminated with EHEC accidentally during or after the slaughtering process. Then, the bacterial cells adhere to the meat tissue and invade to some extent, empowered by an arsenal of attaching and effacing virulence factors. This allows the bacterial cells to grieve more nutrition and escape external extreme conditions, such as disinfection treatment (Bardiau et al., 2010; McWilliams and Torres, 2014; Pradel et al., 2015; Monteiro et al., 2016). For those reasons, countries like Japan and the United States of America strictly regulated raw beef consumption due to the high risk of EHEC contamination. The United States established a zero-tolerance policy against EHEC O157 and six additional serogroups O26, O45, O103, O111, O121, and O145, not allowing a single cell of EHEC in food materials served raw (Forsythe, 2010; USDA, 2010, 2012). However, the risk assessment report, number 691 of the year 2011, of the Food Safety Commission of Japan (FSCJ) adopted a different approach based on some available facts, data calculations, and assumptions. This approach perhaps leads to an extreme level of protection nearly but not exactly equivalent to the zero-tolerance policy in the United States. The FSCJ proposed that a Food Safety Objective (FSO) for EHEC, similarly for *Salmonella* species, must be set at 0.014 CFU/g in raw meat per person portion (50 g of raw meat). Then concluded that setting Performance Objective (PO) at one-tenth (0.0014 CFU/g) of the proposed FSO would be appropriate while taking into account the rations of the pathogens from utensils to meat at cross contaminations and potential growth rate of the pathogens under the assumption that adequate hygienic control measures were implemented. The same report mentioned that because there is no internationally validated detection method for EHEC, a big number of samples are required for direct inspection of pathogenic bacteria. Therefore, *Enterobacteriaceae* are considered an approved indicator of fecal contamination including EHEC and *Salmonella* species. In an additional conclusion in the report, it was mentioned that the PO cannot be met if the number of samples to be tested is 1. In other words, in case of a large amount of meat, the whole lot cannot be judged safe by only testing one portion of the tested meat (weighing 25 g) even if it gives negative results for *Enterobacteriaceae*. While a minimum of 25 samples taken and proven negative can achieve the proposed PO at 97.7% probability (a standard deviation of 1.2 log CFU/g) with 95% confidence. The report finally concluded that although the proposed processing standards only have a certain risk reduction effect, such an effect is not always necessary to achieve an appropriate level of health protection (ALOP). Therefore, the processing standards must be accompanied by microbiological tests to make sure that the PO for raw meat is met (FSCJ, 2011). Practically speaking, when setting up a processing system, including heat treatment, a treatment that guarantees a sufficiently high temperature (60°C for 2 min) to reach inside the tissue of meat (≥1 cm deep) through the surface, will most likely be able to reduce the bacterial count below the ALOP baseline. However, a standard coliform test must be negative for 25 samples (25 g per sample) minimum. Only then, the core part of the meat produced by this treatment would be considered safe for raw consumption, for example, prepared in a raw beef dish known as beef Yukke in Japan (originally known as Yukhoe in Korea and many other parts of the world).

In 2010, we developed a calcium hydroxide–ethanol–lactate-based disinfectant (here and after referred to as CEL disinfectant) composed of natural components: sodium lactate, ethanol, calcium hydroxide, lactic acid, and pure water, thus, harmless to human. It has no effect on the taste and smell of food materials after treatment. The bactericidal effect of the CEL disinfectant itself was determined by a modification of the Kelsey–Sykes method (K-S method) (Fujimoto modification) (Kelsey and Maurer, 1974; Fujimoto, 1982), a test or its modifications have been considered the method of choice to determine the effective concentration of a disinfectant.

In this research, we aimed at developing a method with bactericidal effect and, hypothetically, more nutrition-preserving property that is equivalent to the heat treatment or even superior to meet the PO for raw beef consumption by showing a reduction of viable EHEC cell number (power of reduction) by a factor of 10^4^ CFU or more. We examined the ability of CEL disinfectant to act effectively against EHEC cells contaminating the beef in a combination of physical treatment by two methods, a washing machine disinfection method (WM method) and a vibration-vacuum disinfection (VV) method.

Foodborne infections caused by EHEC are a worldwide issue not only in Japan. Therefore, we are proposing our new WM method and VV method as alternatives to the currently approved heating method as international standard disinfection methods for safe raw beef consumption.

## Materials

### Beef Samples

**FIGURE 1:**
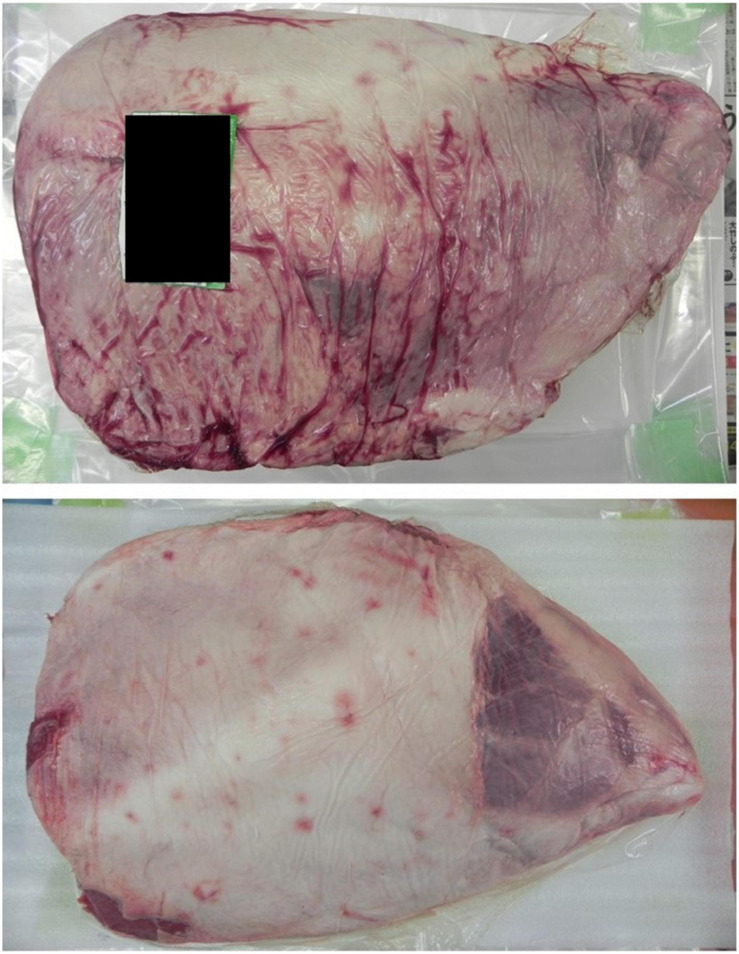
The type of beef (beef leg) used in this research.

All the beef samples we used in this research were supplied by Meat Crest Co., Ltd., and transported within 2–3 days from Oita city to Kyoto city by private logistics companies under cooling conditions. Each sample is a boneless Round-beef requested in full size and covered with its natural fat on the surface (Figure 1).

### Bacterial Strains

We used two of our laboratory stock *E. coli* standard strains. The first one was *E. coli* EDL933, a pathogenic strain characterized by the presence of the *stx* gene (Riley et al., 1983), as a representative of EHEC. The second one was *E. coli* NIHJ, a non-pathogenic strain (Fugono and Maeda, 1977) as a representative of *E. coli* in general; we meant to use it in the slaughterhouse, where EHEC is restricted, in one experiment that implemented artificial contamination and a spray method test.

### Calcium Hydroxide–Ethanol–Lactate Disinfectant

We used a commercially available CEL-based disinfectant made of natural components: sodium lactate 0.54 mol/L, calcium hydroxide 0.035 mol/L, lactic acid 0.0011 mol/L, and ethanol 9.9%; pH 13.0 (15°C) (Shell Coat, previously named as Kinkoros Water, Kawakami Co., Ltd., Osaka, Japan).

### Experiment Sites

We performed all the experiments in the laboratory of the Center for Southeast Asian Studies, Kyoto City, Japan, in a Biosafety Level 2 facility (BSL2), except for one experiment that we carried out at a collaborative slaughterhouse, Meat Crest Co., Ltd., in Oita City, Japan. However, all the other beef leg samples intended for the spray method experiments were sprayed with CEL disinfectant by trained workers in the slaughterhouse before packaging and shipping to the laboratory for further investigations.

### Culture Media

#### Enrichment Media

**LB** (Luria Broth L3522-250G, SIGMA Life Science, SIGMA-ALDRICH, Switzerland); **LB agar** (Luria Broth with added 15 g/L agar powder) and Agar Powder (01028-85 Nacalai Tesque, Kyoto, Japan); **TSB** (Bacto^TM^ Tryptic Soy Broth, Difco, Becton Dickinson Microbiology Systems, Sparks, MD, United States); **BPW** (Buffered Peptone Water, peptone 10 g, sodium chloride 5 g, Na_2_HPO_4_-12H_2_O 9 g, KH_4_PO_4_ 1.5 g, distilled water 1,000 ml, pH 6.8–7.2).

#### Selective Media

**VRBD agar** (Crystal violet neutral-red bile glucose agar; Merck KGaA, Darmstadt, Germany); **CHROMagar STEC** (CHROMagar^TM^ STEC with 50 mg/L STEC supplement, CHROMagar, Paris, France); **BGBB** (Brilliant Green Bile 2% broth, Oxoid Ltd., Hants, United Kingdom).

### Devices

**Pulsifier** (Pulsifier^®^ PUL100, Microgen Bioproducts Ltd., United Kingdom); **Washing machine** (Hitachi BD-V9800L, Hitachi Global Life Solutions Inc., Tokyo, Japan); **Commercially available fitness vibration machine** (SYOSIN RC-CFM-V28B, China); **Micro Diaphragm pumps** [voltage DC 12V, power (max) 60 W, pressure (max) 0.8 Mpa, flow (max) 5 L/min]; **Handheld plastic bag sealer** (Portable sealer FKR-400, Ningbo, China); **Mini Heating Dry Bath Incubator** (MD-MINI/MD-MINI-B01, Major Science, Saratoga, CA, United States).

### Loop-Mediated Isothermal Amplification Kit

EHEC detection by loop-mediated isothermal amplification (**LAMP**) **VT** kit (Loopamp R Verotoxin-producing *Escherichia coli* Detection Kit, Eiken Chemical Co., Ltd., Tokyo, Japan); *Salmonella* detection by **LAMP Sal** kit (Loopamp R *Salmonella* Detection Kit, Eiken Chemical Co., Ltd., Tokyo, Japan).

## Methods

### Scanning Electron Microscope Photos

We prepared beef samples contaminated with the EDL933 strain treated with or without high-speed washing in the CEL disinfectant for 5 min. We submitted these samples to the Graduate School of Medicine at Kyoto University to obtain scanning electron microscope (SEM) photos. The specimens were fixed with 4% paraformaldehyde and 0.05% glutaraldehyde at 4°C for 4 h. After post-fixation with 1% OsO_4_ for 2 h, they were dehydrated using Freeze Dryer ES-2030 (Hitachi Ltd., Tokyo, Japan) and coated with a thin layer of platinum palladium using Ion Coater IB3 (Eiko Corporation, Tokyo, Japan). The specimens were examined with a Hitachi S-4700 SEM (Hitachi Ltd., Tokyo, Japan).

### A Comparative Trial Employing Positive and Negative Controls

We conducted an experiment using beef samples immersed into a bacterial suspension of EHEC EDL933 of 10^5^ CFU/ml for 10 min, a comparative trial using a 0.85% normal saline solution (NSS) as a negative control and a sodium hypochlorite solution 150 ppm as a positive control. Briefly, a piece of 5 g of beef was soaked in 10^5^ CFU/ml culture solution of EHEC EDL933 strain for 10 min at room temperature, then immersed in the CEL disinfectant for a given time. The CEL disinfectant was replaced with controls in a repeated experiment for comparison. Lastly, the piece of beef was immersed in NSS to deactivate the disinfectant. For further assessment of the CEL disinfectant, we have employed a modification of the K-S method (Fujimoto, 1982). The reaction temperature of 20°C was maintained throughout the tests, a universal container is not used in this modification, and 5% of yeast solution was used as an organic substance (dirty conditions). The bacterium in our test was suspended in standard hard water (342 ppm hardness) for the test under clean conditions (no yeasts added). The bacterial suspension was sequentially added (first, second, and third) to each test. After a given exposure time, the mixture was sampled for survivors in a semiquantitative way by inoculating several culture broths.

### Washing Machine Disinfection Method–Artificial Contamination

A Round-beef leg covered with skin fat undergoes four stages, as follows:

#### Stage 1 (No Treatment)

We trimmed the leg at both exposed red edges (two slices, 4–6 cm thick) to obtain the negative control (no artificial contamination) samples. Three pieces of 25 g of negative controls were prepared.

#### Stage 2 (Artificial Contamination)

We soaked the remaining part of the leg in a bacterial suspension of EHEC EDL933 strain (Riley et al., 1983), grown in LB overnight for 10 min at room temperature. Three pieces of 25 g were similarly obtained as positive controls as the negative controls.

#### Stage 3 (Disinfection)

We transferred the contaminated leg to a new container and rinsed for 10 min (pre-disinfection) with 10 L of CEL disinfectant. Then, the leg was transferred again to a commercially available washing machine, Hitachi BD-V9800L, Hitachi Global Life Solutions Inc., Tokyo, Japan. The main disinfection treatment took place inside the machine in three cycles. Each cycle consisted of washing (15 min), rinsing with CEL disinfectant (25 min), then followed by draining of the used disinfectant (10 min).

#### Stage 4 (Post-disinfection)

We transferred the treated leg onto a cutting board. The fat, the skin, and the denatured 1–2 cm depth of the meat surface were trimmed out. Twenty-five samples were collected, 13 and 12 samples, each weighing 25 g, from the two ends and the top central side of the leg, respectively.

#### Microbiological Tests (One-Fifth Method)

Each 25-g sample in every stage was suspended in 225 ml of TSB (Bacto^TM^ Tryptic Soy Broth; Difco, Becton Dickinson Microbiology Systems, Sparks, MD, United States) using a Pulsifier^TM^ PUL100, Microgen Bioproducts Ltd., United Kingdom, for 15 s. Bacterial contamination in the suspensions, of the negative and positive controls, and post-disinfection samples was determined by a plate count method on VRBD agar (crystal violet neutral-red bile glucose agar; Merck KGaA, Darmstadt, Germany) targeting only EHEC and any possible naturally presented coliforms (quantitative technique). Furthermore, an approximately one-fifth (52 ml) of the resultant suspension of each sample was dispensed into 13 tubes (4 ml of the suspension in 36 ml of TSB per tube) (qualitative technique with high sensitivity). All tubes were incubated for 18–24 h at 37°C. Turbid tubes were streaked out on CHROMagar STEC for the detection of EDL933 strain colonies.

### Washing Machine Disinfection Method–Artificial Contamination With EHEC EDL933–Standard Microbiological Tests–Loop-Mediated Isothermal Amplification Method

A Round-beef leg covered with skin fat undergoes the first three stages as described in *Washing Machine Disinfection Method–Artificial Contamination* of the “Methods” section. In the fourth stage, however, a slice of 5–6 cm thick was cut at the middle of the leg, and 25 pieces of 25 g of beef were prepared following a standard method by the Japanese Ministry of Agriculture, Forestry and Fisheries (MAFF, 2012).

#### Microbiological Tests (Standard Method)

Inspection of the coliform in general, and EDL933 in particular, in the negative control, positive control, and post-disinfection samples was carried out qualitatively and quantitatively. First, qualitative detection was performed using a standard method for Enterobacteriaceae detection in raw edible meat approved by the Japanese Ministry of Health, Labour and Welfare (MHLW, 2011); briefly, with minor modifications, each 25-g sample was homogenized and suspended in 225 ml of buffered peptone water (BPW) using the Pulsifier for 15 s. The suspension was incubated overnight at 37°C. One milliliter of the culture was transferred to 10 ml of brilliant green bile broth (BGBB) (Brilliant Green Bile 2% broth, Oxoid Ltd., Hants, United Kingdom) and incubated overnight 37°C. The new culture was streaked onto the VRBD agar plate and incubated overnight at the same temperature. Only three coliform-like (a dark purple/violet colony surrounded by a reddish halo) colonies per plate, if presented, were transferred onto the LB agar plates and incubated overnight at 37°C. Grown colonies were tested for oxidase production and glucose fermentation. The result of this standard method was compared with the result of the LAMP method for both *E. coli* and *Salmonella* as described below. Second, quantitative detection was performed in the sample suspension in BPW using a plate count method on VRBD agar.

#### Loop-Mediated Isothermal Amplification Method

DNA template solutions of the BPW and the BGBB enriched cultures were extracted by a boiling method for the *stx* gene detection by the commercially available LAMP kit of verotoxin-producing *E. coli* detection as instructed by the manufacturer and previously described by Kayali et al. (2015). Exceptionally, the presence of *Salmonella* spp. was investigated by the LAMP method using a commercially available detection kit for *Salmonella* spp. because of its importance from a health concern standpoint. LAMP reactions were carried out in a mini heating dry bath incubator that guarantees stable amplifications at a temperature of 60°C for 60 min, followed by inactivation at a temperature of 80°C for 5 min. The positive result of LAMP was judged with the naked eyes by observing turbidity in the reaction tube. No turbidity in the tube meant a negative result of LAMP.

### Spray Method–*Escherichia coli* NIHJ Strain

An overnight culture of a non-pathogenic *E. coli*, NIHJ strain (Fugono and Maeda, 1977), grown in LB at 37°C, was used to contaminate 10 cm^2^ of the exposed red meat area, on the surface of two Round-beef legs of the same cattle, by swabbing. Immediately after contamination, three distinct 1-cm^2^ spots within every 10 cm^2^ were swabbed (positive control at 0 min). Swabs were suspended in sterile 9 ml of 0.85% NaCl. One leg was sprayed with approximately 300 ml of CEL disinfectant, while the other leg was kept without spray. Swabbing was repeated after 3 days of transportation to the laboratory at 4°C, consecutively, in the same manner as described earlier for comparison. The number of bacterial cells of NIHJ strain in each swab suspension was determined by the plate count method on VRBD agar. Finally, both legs were independently disinfected by the WM method and microbiologically inspected as previously described in the WM method. Besides, the synergistic power of reduction (Synergistic PoR) was calculated by adding the PoR of the spray method, as a potential reduction factor when applied to the observed PoR of any of the physical treatments, like the WM method or the VV method that is described later.


SynergisticPoR=ObservedPoRbyaphysicalmethod+

PotentialPoRbytheSpraymethod

PoR=PositiveControl(CFU/ml)-Posttreatment(CFU/ml)

### Spray Method–*Escherichia coli* EDL933 Strain

The effect of the same spray method, used with NIHJ strain, was investigated this time with *E. coli* EDL933 strain in the BSL2 by following the same steps with minor modifications, such as using relatively smaller blocks of beef (around 0.5 kg each).

### Spray and Washing Machine Disinfection Methods–No Artificial Contamination–Standard Microbiological Tests–Loop-Mediated Isothermal Amplification Method

The spray method and the WM method combined were applied on a beef leg without artificial contamination. We compared the effect of the spray method with (W/) or without (W/o) the CEL disinfectant spray. We requested the meat manufacturer to prepare three distinctive cuts from the beef leg sample before spraying the CEL disinfectant (W/o spray samples). The rest of the leg was sprayed with the disinfectant, packaged, and shipped to the laboratory. In the laboratory, we prepared another three distinctive cuts from the big part of the beef leg at the same positions of the W/o spray samples (those to be designated as W/spray samples). The rest of the leg was treated with the WM method as described above. Microbiological tests following the standard method were done in addition to LAMP tests as described in *Washing Machine Disinfection Method–Artificial Contamination With EHEC EDL933–Standard Microbiological Tests–Loop–Mediated IsothermalAmplification Method* of the “Methods” section.

### Vibration-Vacuum Disinfection Method–Artificial Contamination With EHEC EDL933–Standard Microbiological Tests–Loop-Mediated Isothermal Amplification Method

Round-beef leg covered with skin fat was processed in four stages, as follows:

#### Stage 1 (No Treatment)

The leg was trimmed at three exposed red meat corners (three cuts, about 6 cm thick) to obtain the negative control (no artificial contamination). Three pieces of 25 g of negative controls were prepared.

#### Stage 2 (Artificial Contamination)

The remaining part of the leg was soaked in a bacterial suspension of EHEC EDL933 strain, grown in LB overnight, at a concentration of 3.3 × 10^7^ CFU/ml for 10 min at room temperature. Three pieces of 25 g were similarly obtained as positive controls as the negative controls.

#### Stage 3 (Disinfection)

The remaining part of the contaminated leg was placed inside an exclusively designed plastic bag and sealed by a commercially available handheld sealer (Portable sealer FKR-400, Ningbo, China). The sealed bag was put in a strong zipped-net prepared out of a fishing net and shaped like the beef leg. The zipped-net was slightly bigger to allow some room for CEL disinfectant to spread around the beef and hold the pressure inside the plastic bag. Next, the ready bag with beef was placed on a tray on top of a commercially available fitness vibration machine (SYOSIN RC-CFM-V28B, China). Another plastic bag filled with around 17 L of water (the volume of water could be more or less depending on the overall weight of the beef leg and CEL disinfectant inside the beef bag) was used as a balancer beside the beef bag on top of the machine. Then, the ready beef-in-bag was connected to a network of tubing, one tubing line that supplies fresh CEL disinfectant and two tubing lines that suck up or discard the used disinfectant from the bag. The CEL disinfectant was introduced by a series of three Micro Diaphragm pumps (three-diaphragm pump-series), voltage DC 12 V, power (max) 60 W, pressure (max) 0.8 Mpa, flow (max) 5 L/min through one tube of 8-mm diameter with a check (one-way) valve; however, the CEL disinfectant was sucked up by two parallel three-diaphragm pump-series through two 6-mm-diameter tubes, each had a filter-end inside the bag and consisted of a check valve too. The disinfection procedure was as follows: four rounds of positive supply with up to 5 L of fresh CEL disinfectant then vibration for 10 min at 860 rpm, followed by vacuum while vibrating at 315 rpm for around 10 min at the end of each round. Then, there were three rounds of positive supply with up to 8 L of fresh CEL disinfectant, vibration for 10 min at 860 rpm, followed by vacuuming while vibrating at 315 rpm for around 7 min, then rinsing with around 2 L of the CEL disinfectant until the complete vacuum with a continuous vibration at 315 rpm for approximately 5 min.

#### Stage 4 (Post-disinfection)

The bag was disinfected externally with 70% ethanol. The treated leg was transferred carefully from the bag to a cutting board. A slice of 5–6 cm thick was cut in the middle of the leg, and 25 pieces of 25 g of beef were prepared following a standard method by the Japanese Ministry of Agriculture, Forestry and Fisheries (MAFF, 2012).

#### Microbiological Tests (Standard Method)

The microbiological tests following the standard method were as described above in *Washing Machine Disinfection Method–Artificial Contamination With EHEC EDL933–Standard Microbiological Tests–Loop-Mediated Isothermal Amplification Method*. Also, the sample suspensions in BPW were cultured on VRBD agar.

### Spray and Vibration-Vacuum Disinfection Method–No Artificial Contamination

The spray method and the VV method combined were tested with beef leg without artificial contamination. The presence or absence of EHEC and *Salmonella* spp. in the enriched BPW and BGBB cultures was confirmed by LAMP.

### Ca Concentration

The concentration of calcium in treated meat with or without CEL disinfectant was investigated at the surface (1–2 cm deep) and in the internal part of the meat (2–4 cm deep).

### Disclaimer

All the experiments were done in triplicate, and averages were shown, except in cases where more than one experiment was very inconvenient or one experiment was considered sufficient to serve the intended purpose. As a safety procedure, all experiments where live EHEC EDL933 was utilized or believed to be existing or remained were done inside the BSL2 or in well-closed systems, which allowed an overall control of these harmful bacteria.

## Results and Discussion

### Scanning Electron Microscope Photos

**FIGURE 2:**
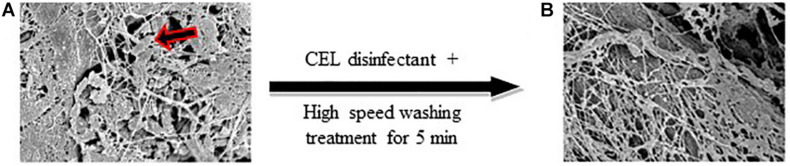
**(A)** Pure culture of EDL933 strain cells (arrow) adhering to the surface of the meat. **(B)** The bacterial cells were detached, while the fibrous structure of the meat remained (×5,000).

The photos revealed that EHEC cells can adhere firmly and form a biofilm-like structure on the surface of the red fibers of the meat (Figure 2A), however, they could barely be found on the fat (data not shown). Comparatively, SEM photos of high-speed washing-treated samples showed that the areas covered with fat were completely cleaned out. This suggested that fat protects the meat against bacterial attachment or invasion and made us not only less concerned about those areas but also request the meat supplier to keep as much fat as possible while preparing the beef legs for us. On the other hand, SEM photos of fibrous areas (exposed red meat) showed that the bacterial cells were detached or washed out without damaging the fibrous structure (Figure 2B). Therefore, we turned our attention completely toward EHEC detection in the red meat area where the meat fibers are exposed.

### A Comparative Trial Employing Positive and Negative Controls

**FIGURE 3:**
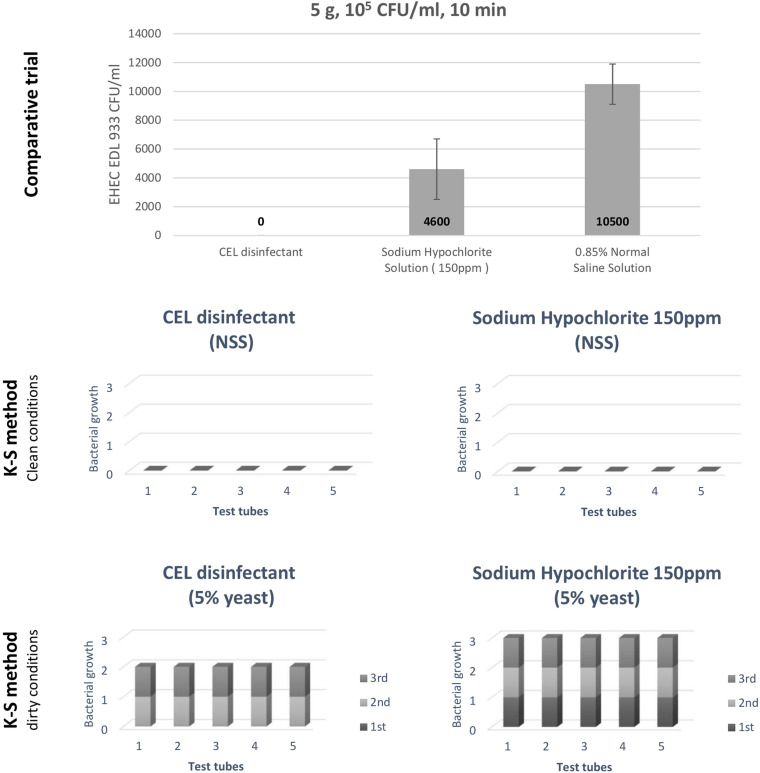
A comparative trial to assess the calcium hydroxide–ethanol–lactate (CEL) disinfectant, and an evaluation of the CEL disinfectant by Kelsey–Sykes (K-S) method (against EHEC *Escherichia coli* O157:H7 EDL933).

In this comparative trial, we examined the bactericidal effect of CEL disinfectant in comparison with sodium hypochlorite and NSS against EHEC EDL933. After treatment for 10 min, the survivability of the inoculated bacteria was examined by swabbing onto VRBD agar plates. No growth was indicated on plates of samples treated with CEL disinfectant. Average CFU/ml was calculated for the samples treated with sodium hypochlorite solution 150 ppm and compared to the ones treated with only NSS (Figure 3, top). It clearly showed a stronger effect of the CEL disinfectant. However, the level of strength was investigated following the K-S method (Fujimoto modification). Both CEL disinfectant and sodium hypochlorite showed similar bactericidal effects in clean conditions (NSS). However, CEL disinfectant was shown to be superior to sodium hypochlorite in dirty conditions (5% yeast) only when the first bacterial suspension was added. In conclusion, the results showed that the bactericidal effects of both CEL disinfectant and sodium hypochlorite decreased in dirty conditions. Nevertheless, even under such a condition, the effect of CEL disinfectant was confirmed to be higher than that of sodium hypochlorite (Figure 3, middle and bottom). On the other hand, CEL disinfectant efficacy reduction by the presence of organic materials indicated that the presence of the beef residue during the disinfection process would have a similar impact. We conducted some series of trial experiments and found it true (data not shown). However, we noticed that a gradual use of a physical motion during the disinfection process as well as a continuous replacement of the used disinfectant with a fresh equivalent volume helped boost the effect of the CEL disinfectant against the bacteria with a little denaturation of the beef surface (data not shown). Therefore, a physical treatment was adopted into our developed disinfection methods, the WM method and the VV method.

### Washing Machine Disinfection Method–Artificial Contamination

**FIGURE 4:**
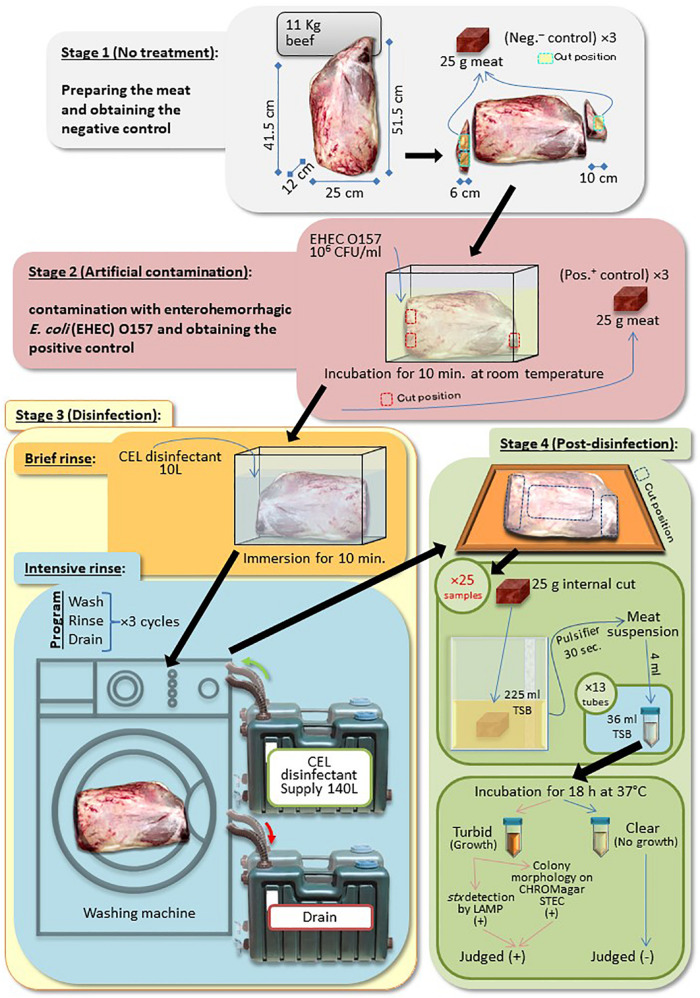
A summarized protocol of the washing machine disinfection (WM) method.

The WM method is summarized in Figure 4. The result by plate count method of three experiments [beef leg weight 12.1 ± 0.5 kg; EHEC (EDL933) contaminating culture suspension 1.1 ± 1.4 × 10^8^ CFU/ml] showed that the WM method effectively reduced the bacterial load by a factor of 10^5^ CFU/ml. Whereas the one-fifth method showed that the percentage of the number of EHEC-positive tubes per sample was reduced from 100 to 21.5% (Table 1). This physical treatment took around 2.5 h; however, it consumed a large amount of the disinfectant (150 L).

**TABLE 1:** The result of artificially contaminated beef with EDL933 treated by WM method.

Detection	By plating (CFU/ml)	By turbidity (one-fifth method)	
	Average	Average (%)	
Post-infection (Positive control)	2.7 ± 2.2 × 10^5^	13 ± 0 (100)	EHEC^Pos+^ (*n* = 13)
Post-disinfection (WM method)	1.8 ± 3.1 × 10^2^	2.8 ± 1.3 (21.5)	EHEC^Pos+^ (*n* = 13)
Power of reduction (PoR)	1.5 ± 0.7 × 10^5^	10.2 ± 11.7 (78.5)	EHEC^Neg–^ (*n* = 13)

*WM, washing machine disinfection.*

Although our result showed an important reduction of EHEC bacterial cell count that meets with the proposed PO by the FSCJ in Japan, it was essential to prove the high level of reduction by examining a large number of samples as the FSCJ recommended in its report. In our protocol, we designed a sensitive and less time-consuming microbiological test than the standard one for EHEC detection in beef. We designated it as the one-fifth method. Both methods examine 25 samples; our method took 1–3 days to confirm the result, while the standard method took 3–7 days. However, we found it mandatory to apply the standard detection method for Enterobacteriaceae to evaluate our main disinfection method in a standard way for a better judgment. Therefore, we replaced the one-fifth method with the standard coliform test method. Applying the two methods at the same time was a big hassle, so we avoided that.

### Washing Machine Disinfection Method–Artificial Contamination With EHEC EDL933–Standard Microbiological Tests–Loop-Mediated Isothermal Amplification Method

In this experiment, where we used one beef leg (12.7 kg) and contaminated it with 4.4 × 10^7^ CFU/ml of EDL933 culture suspension, we confirmed that the PoR of the WM method was higher than 10^4^ CFU/ml, which was shown to be 0.5 × 10^5^ CFU/ml. However, the result of the standard coliform test, which we applied here, was not very satisfactory. Thus, we tried to introduce the LAMP method as a more specific technique that examines a narrow group of bacteria like verotoxin-producing *E. coli* and *Salmonella* spp. rather than examining the entire family of Enterobacteriaceae (Table 2).

**TABLE 2:** The result of one artificially contaminated beef with EDL933 treated by the WM method.

Detection	By plating on VRBD^a^ (CFU/ml)			
Post-infection (Positive control)	3.1 × 10^5^		
Post-disinfection (WM method)	6.7 × 10^0^		
Power of reduction (PoR)	0.5 × 10^5^		

	**Standard microbiological tests**		**LAMP method**	
			
	**Growth on VRBD**	**Coliform test (Standard method)**	**LAMP test for BGBB culture^b^**	

			**VT^c^**	**Sal^d^**
Negative control (No treatment) (*n* = 3)	0:3^e^	0:3	3:0	3:0
Positive control (Post-infection with EHEC) (*n* = 3)	0:3	0:3	0:3	3:0
Post-disinfection (WM method) (*n* = 25)	1:24	1:24	1:24	25:0

*^a^VRBD: Violet Red Bile Dextrose agar. ^b^DNA template solutions were obtained from the second enrichment culture BGBB; LAMP test showed no false-positive result. ^c^VT: verotoxin- producing E. coli detection LAMP. ^d^Sal: Salmonella detection LAMP. ^e^Negative to positive ratio. LAMP, loop-mediated isothermal amplification; WM, washing machine disinfection.*

On one hand, it was noticeable that the negative control gave a positive result by the coliform test for three tested samples; however, the LAMP VT and LAMP Sal tests were negative for the same samples. We concluded here that the LAMP method excluded unnecessary concerns of *E. coli* or *Salmonella* spp. due to coliform false-positive test results due to other kinds of bacteria. On the other hand, the LAMP tests of the first enrichment in BPW showed some false-positive results, but not in the second enrichment in BGBB. This could be due to several reasons such as the presence of unknown components in the culture. Therefore, testing the DNA template solutions prepared out of the second enrichment (BGBB) could be the right option. Finally, the result by the coliform test could be obtained after 5 consecutive days, while the result by the LAMP could be ready after 1 day, which significantly reduces time and labor.

### Spray Method–*Escherichia coli* NIHJ Strain

The result of the WM method was very encouraging; however, we sought additional direct or indirect improvement. It is important to remember that meat contamination might happen at some early stage during slaughtering or manufacturing. For this reason, we visited the meat source headquarters to investigate the result of applying the minimum disinfection of beef during manufacturing.

This experiment was conducted in the slaughterhouse. The pathogenic strain EDL933 was not permitted, therefore, it was replaced with the non-pathogenic strain NIHJ. Two beef legs (11.2 ± 0.2 kg) were used and artificially contaminated with 3.3 × 10^8^ CFU/ml of NIHJ culture suspension. The number of viable cells of NIHJ strain decreased within 10 min after CEL disinfectant spray treatment (approximately 300 ml) from 5.2 × 10^5^ CFU/cm^2^ to 2.6 × 10^4^ CFU/cm^2^. Moreover, the reduction was still observable from 5.2 × 10^5^ CFU/cm^2^ to 4.0 × 10^4^ CFU/cm^2^ after 3 days of storage and transportation at 4°C. However, by comparing the sample that was kept without spray under the same storage conditions for 3 days, the cell count showed no reduction but slightly increased. As a result, EHEC adhering to the meat could be reduced more than 10^5^ times after including **the synergistic power of reduction (Synergistic PoR)**, adding up the PoR of the spray method to the PoR of the second stage of disinfection (physical treatment) in the laboratory (Table 3).

**TABLE 3:** Effect of the spray method on NIHJ strain (non-EHEC), artificially contaminating two beef legs, and the synergistic effect of both spray and WM methods.

T-point	Detection	By plating		By turbidity (one-fifth method)		
		W/o spray^a^	W/spray ^b^	W/o spray	W/spray	
0 min	Post-infection (CFU/cm^2^)	5.2 × 10^5^	5.2 × 10^5^	13	13	NIHJ^Pos+^ (*n* = 13)
3 days	After transportation (CFU/cm^2^)	8.6 × 10^5^	4.0 × 10^4^			
	Power of reduction (times)	n/a^c^	1.3 × 10^1^			
	Potential power of reduction^d^	n/a	2.15 × 10^1^			
	Post-disinfection (WM method) (CFU/ml)	0	0	0	0	NIHJ^Pos+^ (*n* = 13)
	Power of reduction (PoR)	8.6 × 10^5^	5.2 × 10^5^	13 (100%)	13 (100%)	NIHJ^Neg–^ (*n* = 13)
	Synergistic power of reduction^e^	n/a	1.1 × 10^7^			

*^a^W/o: without. ^b^W/: with. ^c^n/a: not applicable. ^d^Potential power of reduction of spray method = without spray count − with spray count. ^e^Synergistic power of reduction = observed power of reduction + potential power of reduction. WM, washing machine disinfection.*

### Spray Method–*Escherichia coli* EDL933 Strain

The same spray method was tested with EDL933 strain in the laboratory. The result showed a relatively similar effect as shown with NIHJ strain (Table 4). The beef samples contaminated with 9.9 × 10^7^ CFU/ml of EDL933 culture suspension were sprayed with CEL disinfectant. The result showed a PoR of 2.1 × 10^1^ CFU/cm^2^ after 3 days of applying the spray. We designated this result as the **Spray method PoR factor** that should be considered and combined with the PoR of the physical treatment, as the WM method, to achieve and guarantee the highest disinfection level. Therefore, it would be recommended that the spray method is adapted during meat production as a simple but critical step.

**TABLE 4:** The effect of the spray method on the viability of EDL933 strain cells.

T-point	Detection	By plating (CFU/ml)
		W/spray^a^
0 min	Post-infection (CFU/cm^2^)	2.8 ± 1.5 × 10^5^
3 days	Storage period (CFU/cm^2^)	9.8 ± 3.1 × 10^3^
	Power of reduction (times)	2.1 ± 4.2 × 10^1^

*^a^W/: with.*

### Spray and Washing Machine Disinfection Methods–No Artificial Contamination–Standard Microbiological Tests–Loop-Mediated Isothermal Amplification Method

**FIGURE 5:**
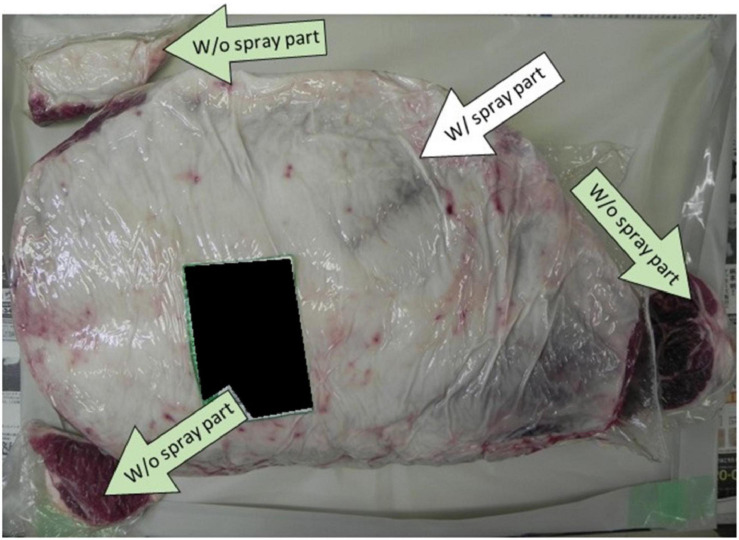
Showing the positions where the spray method with calcium hydroxide–ethanol–lactate (CEL) disinfectant was applied on the beef leg.

In this experiment, we studied the effect of synergistic use of both the spray method (by comparing W/o spay and W/spray samples side by side) (Figure 5) and the WM method on the beef (right side leg 12.5 kg) prepared regularly at the slaughterhouse without artificial contamination. The coliform presence was tested following the recommended standard method. The result showed a 100% elimination of the coliforms that were originally presented in the beef (Table 5). LAMP results showed no detection of EHEC nor *Salmonella* in comparison with the negative and positive controls.

**TABLE 5:** Summary of spray method and WM method synergistic effect on the beef without artificial contamination.

	Standard microbiological tests		LAMP method	
	Growth on VRBD^a^	Coliform test (standard method)	LAMP test for BGBB culture^b^	
			**VT^c^**	**Sal^d^**
W/o spray (*n* = 3)	0:3^e^	2:1	3:0	3:0
W/spray (*n* = 3)	2:1	2:1	3:0	3:0
Post-disinfection (WM method) (*n* = 25)	25:0	25:0	25:0	25:0

*^a^VRBD: Violet Red Bile Dextrose agar. ^b^DNA template solutions were obtained from the second enrichment culture BGBB; LAMP test showed no false-positive result. ^c^VT: verotoxin- producing E. coli detection LAMP. ^d^Sal: Salmonella detection LAMP. ^e^Negative to positive ratio. LAMP, loop-mediated isothermal amplification; WM, washing machine disinfection.*

This experiment showed the importance of applying the spray method as well as the LAMP method. The former one minimized the bacterial contamination on the beef, and the latter one excluded the presence of both targets, EHEC and *Salmonella*.

### Vibration-Vacuum Disinfection Method–Artificial Contamination With EHEC EDL933–Standard Microbiological Tests–Loop-Mediated Isothermal Amplification Method

**FIGURE 6:**
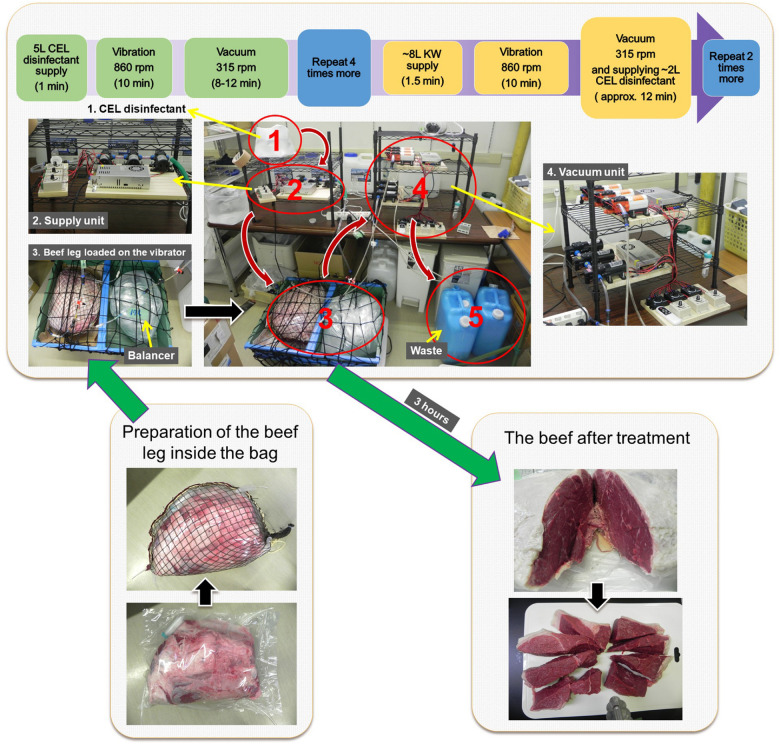
A summarized protocol of the vibration-vacuum disinfection (VV) method.

The VV method is summarized in Figure 6. In this experiment of the improved method, we used one beef leg 12.8 kg artificially contaminated with 3.3 × 10^7^ CFU/ml of EDL933 culture suspension. Although only 50 L of the CEL disinfectant were used during 3 h disinfection treatment, the result showed a Synergistic PoR against EDL933 cells as high as 1.1 × 10^5^ CFU/ml. The microbiological test results of the standard coliform detection method in comparison with the LAMP test results are shown in Table 6.

**TABLE 6:** The result of one artificially contaminated beef with EDL933 treated with the VV method.

Detection	By plating on VRBD^a^ (CFU/ml)			
Post-infection (Positive control)	8.1 × 10^4^		
Post-disinfection (WM method)	1.7 × 10^1^		
Power of reduction (PoR)	0.5 × 10^4^		
Spray method PoR factor	2.1 × 10^1^		
Synergistic PoR	1.1 × 10^5^		

	**Standard microbiological tests**		**LAMP method**	
	**Growth on VRBD**	**Coliform test (standard method)**	**LAMP test for BGBB culture^b^**	
			**VT^c^**	**Sal^d^**

Negative control (no treatment) (*n* = 3)	0:3^e^	0:3	3:0	3:0
Positive control (post-infection with EHEC) (*n* = 3)	0:3	0:3	0:3	3:0
Post-disinfection (WM method) (*n* = 25)	0:25	0:25	0:25	25:0

*^a^VRBD: Violet Red Bile Dextrose agar. ^b^DNA template solutions were obtained from the second enrichment culture BGBB; LAMP test showed no false-positive result. ^c^VT: verotoxin- producing E. coli detection LAMP. ^d^Sal: Salmonella detection LAMP. ^e^Negative to positive ratio. LAMP, loop-mediated isothermal amplification; VV, vibration-vacuum disinfection; WM, washing machine disinfection.*

The result of the VV method showed that it could meet the proposed PO of the FSCJ in economical manners by scaling down the use of CEL disinfectant by one-third.

### Spray and Vibration-Vacuum Disinfection Method–No Artificial Contamination

We studied the effect of synergistic use of the spray and VV methods on three beef legs prepared at the slaughterhouse, with or without the spray method, with no artificial contamination. The coliform presence was tested following the recommended standard method. The result showed 100% elimination of the coliforms that were originally present in the beef. Also, among the three beef leg samples, only one sample was tested by the LAMP method (Table 7).

**TABLE 7:** The result summary of three experiments of spray method and VV method synergistic effect on the beef without artificial contamination.

	Standard microbiological tests			LAMP method (a result of only one experiment)	
	Growth on VRBD^a^	Coliform test (standard method)		LAMP test for BGBB culture^b^	
				**VT^c^**	**Sal^d^**
W/o spray (*n* = 9)	1:8^e^	3:6	(*n* = 3)	3:0	3:0
W/spray (*n* = 9)	3:6	4:5	(*n* = 3)	3:0	3:0
Post-disinfection (WM method) (*n* = 75)	75:0	75:0	(*n* = 25)	25:0	25:0

*^a^VRBD: Violet Red Bile Dextrose agar. ^b^DNA template solutions were obtained from the second enrichment culture BGBB; LAMP test showed no false-positive result. ^c^VT: verotoxin- producing E. coli detection LAMP. ^d^Sal: Salmonella detection LAMP. ^e^Negative to positive ratio. LAMP, loop-mediated isothermal amplification; VV, vibration-vacuum disinfection.*

A similar conclusion could be as previously mentioned about the important role of the spray method and LAMP method.

### Ca Concentration

CEL disinfectant is a calcium hydroxide-based food disinfectant. Therefore, we investigated the concentration of Ca in the treated meat in comparison with non-treated meat. The result showed that Ca concentration in the internal portion of the treated meat was increased by around 76% (Table 8).

**TABLE 8:** The average concentration of calcium in treated meat with or without CEL disinfectant.

Beef W/or W/o treatment^a^	External	Internal
W/o (mg/100 g)	4.5 ± 0.25	4.2 ± 0.17
W/(mg/100 g)	67.1 ± 16.4	7.4 ± 1.2
CEL disinfectant (% = ×1,000 mg/100 ml)	0.127 ± 0.005	

*^a^W/: with, W/o: without. CEL, calcium hydroxide–ethanol–lactate.*

The question is whether this increase in Ca concentration must be considered or not; the Food and Nutrition Board (FNB) at the Institute of Medicine of the National Academies of the United States prescribed a recommended daily intake allowance of calcium ranging from 700 up to 1,300 mg for people between 1 and 71+ years old (Ross et al., 2011). According to our result of calcium concentration, 100 g of raw treated beef a day (like the beef Yukke dish) would contain around 7.4 mg only. Even though we multiply the amount of consumed raw beef a day by 10 times (1,000 g), the daily intake would be 74 mg, and it is still around 10 times below the recommended daily intake of calcium. Nevertheless, it would be recommended that the concentration of calcium, of beef disinfected with CEL disinfectant, is mentioned as a nutrition fact on the beef product for the consumers.

## Conclusion

We used a safe food disinfectant in combination with physical treatment to disinfect contaminated raw beef successfully without affecting the quality of the internal edible part of the meat mass. The disinfection methods (WM method or VV method) supported with microbial detection were used to prove the efficacy of the disinfection treatment of eliminating the target group of bacteria or reduce it to meet the OP standard. It is important to mention that contaminated beef with a high concentration of bacteria (≥10^6^ CFU/ml) is difficult to be completely disinfected. Therefore, we would recommend applying the disinfection by the spray method immediately during meat dressing in the factory to slow down bacterial proliferation, followed by full disinfection treatment (MW method or VV method) as early as possible (within 3–4 days). Although the WM method and VV method both showed a reliable effect against EDL933 by reducing the bacterial load by the factor of 10^4^–10^5^ CFU/ml, we still recommend the synergistic use of the spray method with either of them for the previously mentioned reason. The WM method and VV method are two available options with some differences, as shown in Table 9, which are also suitable with various kinds of industrial economy and labor-wise purposes, etc. We suggest including the LAMP method for sensitive and specific detection of EHEC and *Salmonella* spp. instead of the current standard coliform (Enterobacteriaceae) detection method. Calcium concentration increase in the edible meat treated with the CEL disinfectant is still in a very safe range for human daily intake allowance.

**TABLE 9:** A comparison between the WM method and the VV method.

Method	WM	VV
Time	2.5 h	3 h
Equipment required	Less	More
Disinfectant consumption	150 L	50 L
PoR	Up to 10^5^ CFU/ml	Up to 10^4^ CFU/ml
Synergistic PoR	Up to 10^7^ CFU/ml	Up to 10^5^ CFU/ml
Overall cost	Higher	Cheaper

*PoR, power of reduction; VV, vibration-vacuum disinfection; WM, washing machine disinfection.*

## Data Availability Statement

The original contributions presented in the study are included in the article/supplementary material, further inquiries can be directed to the corresponding author/s.

## Author Contributions

MN and JO conceived the idea of starting the project. MN and AK performed the experiments, and jointly interpreted and analyzed the data. AK wrote the manuscript. JO provided logistic and technical contributions. All authors contributed to the article and approved the final version.

## Conflict of Interest

JO is the head of T. K. Shin Co., Ltd. The T. K. Shin Co., Ltd. had the following involvement with the study: partial financial support for setting laboratory equipment and hiring laboratory staff. MN owns patents US9060541B2, EP2446755B1, CN102802448B, AU2010263554B2, JP4681693B2, and WO2010150850A1 of the sterilizer for foods. MN and JO jointly have patents pending for JP2018157770A and JP2019170020 of methods for producing sterilized meat chunks. All laboratory work and evaluations were undertaken at the laboratory of the Center for Southeast Asian Studies, Kyoto University. The remaining authors declare that the research was conducted in the absence of any commercial or financial relationships that could be construed as a potential conflict of interest.
