# Corrigendum: Development and Improvement of Methods to Disinfect Raw Beef Using Calcium Hydroxide–Ethanol–Lactate-Based Food Disinfectant for Safe Consumption

**DOI:** 10.3389/fmicb.2021.773509

**Published:** 2021-11-09

**Authors:** Ahmad Yaman Kayali, Jo Ozawa, Mitsuaki Nishibuchi

**Affiliations:** ^1^Division of Environmental Coexistence, Center for Southeast Asian Studies, Kyoto University, Kyoto, Japan; ^2^T. K. Shin Co., Ltd., Hyogo, Japan

**Keywords:** raw beef, disinfection, method, enterohemorrhagic *Escherichia coli*, *Salmonella*, calcium hydroxide–ethanol–lactate (CEL) disinfectant, detection, loop-mediated isothermal amplification

In the original article, there were several errors. Firstly, there was a typographical error in the title: “*Development and Improvement of Methods to Disinfect Raw BeefUsing Calcium Hydroxide–Ethanol–Lactate-Based Food Disinfectant for Safe Consumption.”*

The title is now corrected to: “*Development and Improvement of Methods to Disinfect Raw Beef Using Calcium Hydroxide–Ethanol–Lactate-Based Food Disinfectant for Safe Consumption.”*

In Figure 1 and 5 of the original article, the beef sample label sticker was not obscured. The corrected Figure 1 and 5 appear below.

**Figure 1:**
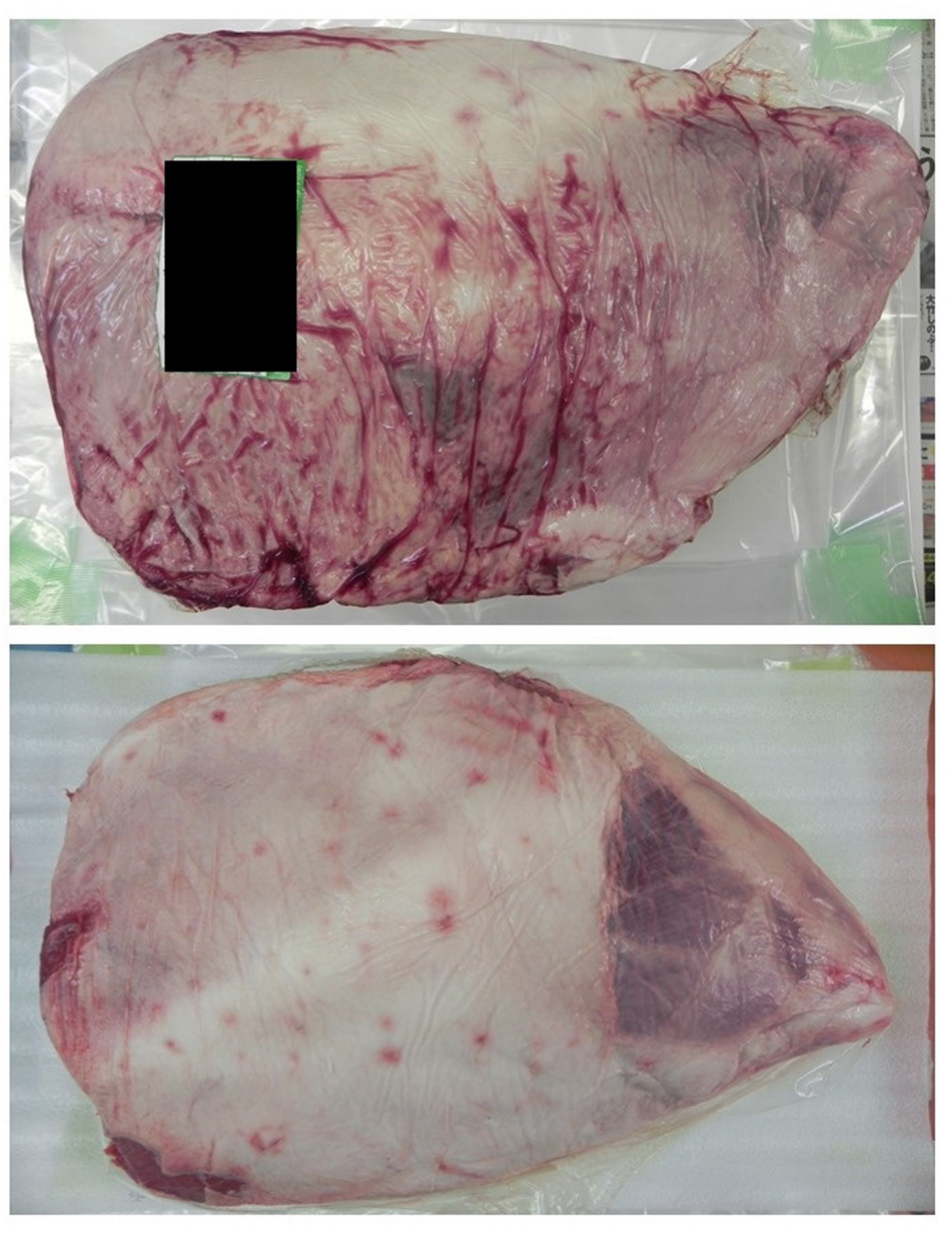
The type of beef (beef leg) used in this research.

**Figure 5:**
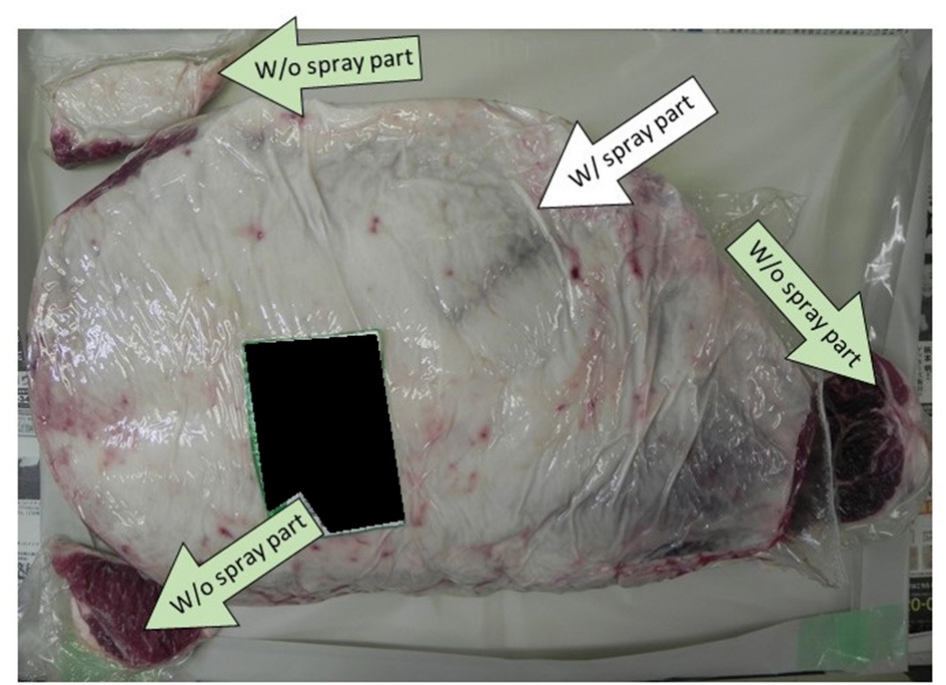
Showing the positions where the spray method with calcium hydroxide–ethanol–lactate (CEL) disinfectant was applied on the beef leg.

Three authors were incorrectly included in the author list: Yasuharu Yamashita^2^, Hiroo Kawakami^2^, and Hironori Kiyota^3^. The correct author list is as follows: Ahmad Yaman Kayali^1*^, Jo Ozawa^2^, and Mitsuaki Nishibuchi^1^^*†*^.

To reflect the removal of these three authors, the affiliations list, Author Contributions section, Conflict of Interest statement, and Acknowledgments section require updating.

The affiliations of the three former authors have been removed. The correct affiliation list is as follows: “^1^ Division of Environmental Coexistence, Center for Southeast Asian Studies, Kyoto University, Kyoto, Japan, ^2^ T. K. Shin Co., Ltd., Hyogo, Japan.”

In the original article, the **Author Contributions** section read: “AK and MN conceived and designed the experiments, performed the experiments, and analyzed the data. YY, HKa, HKi, and JO provided logistic and technical contributions. AK interpreted and wrote the manuscript. All authors contributed to the article and approved the submitted version.”

The correct **Author Contributions** section is as follows: “MN and JO conceived the idea of starting the project. MN and AK performed the experiments, and jointly interpreted and analyzed the data. AK wrote the manuscript. JO provided logistic and technical contributions. All authors contributed to the article and approved the final version.”

In the original article, the **Conflict of Interest** statement read: “YY and HKa were employees of Kawakami Co., Ltd. HKi was an employee of Meat Crest Co., Ltd. JO was an employee of T. K. Shin Co., Ltd. All laboratory work and evaluation were undertaken at the laboratory of the Center for Southeast Asian Studies, Kyoto University. No financial support was provided by Kawakami Co., Ltd., Meat Crest Co., Ltd., and T. K. Shin Co., Ltd. to conduct this study. The remaining authors declare that the research was conducted in the absence of any commercial or financial relationships that could be construed as a potential conflict of interest.”

The correct **Conflict of Interest** statement as follows: “JO is the head of T. K. Shin Co., Ltd. The T. K. Shin Co., Ltd. had the following involvement with the study: partial financial support for setting laboratory equipment and hiring laboratory staff. MN owns patents US9060541B2, EP2446755B1, CN102802448B, AU2010263554B2, JP4681693B2, and WO2010150850A1 of the sterilizer for foods. MN and JO jointly have patents pending for JP2018157770A and JP2019170020 of methods for producing sterilized meat chunks. All laboratory work and evaluations were undertaken at the laboratory of the Center for Southeast Asian Studies, Kyoto University. The remaining author declares that the research was conducted in the absence of any commercial or financial relationships that could be construed as a potential conflict of interest.”

In the original article, the **Acknowledgments** section read: “This research is dedicated to Emeritus Mitsuaki Nishibuchi. We thank Uraiwan Thongchankeaw-Seo, Koji Seo, Sutima Preeprem, Jetnapang Kongrueng, Ryusei Furuya, Takaki Kai, Kaori Murakami, Isao Nakamoto, and Wakana Nakamura for their technical assistance. We also thank Keiko Okamoto-Furuta and Haruyasu Kohda of the Division of Electron Microscopic Study, Center for Anatomical Studies, Graduate School of Medicine, Kyoto University, for technical assistance in electron microscopy. In addition, we thank Fadi Alrouh of the Organic Chemistry Department, Faculty of Medicine, University of Al-Hawash, Syria, for his assistance. Finally, we thank Wataru Yamazaki of the Center for Southeast Asian Studies, Kyoto University for his critical comments on the manuscript.”

The correct **Acknowledgments** section is as follows: “This research is dedicated to Professor Emeritus Mitsuaki Nishibuchi. We thank Yasuharu Yamashita, Hiroo Kawakami, and Hironori Kiyota for their logistic support, Uraiwan Thongchankeaw-Seo for her technical assistance, and Keiko Okamoto-Furuta and Haruyasu Kohda of Graduate School of Medicine, Kyoto University for their technical assistance in electron microscopy. We also thank Wataru Yamazaki of the Center for Southeast Asian Studies, Kyoto University for his critical comments on the manuscript.”

The authors apologize for these errors and state that this does not change the scientific conclusions of the article in any way. The original article has been updated.

## Publisher's Note

All claims expressed in this article are solely those of the authors and do not necessarily represent those of their affiliated organizations, or those of the publisher, the editors and the reviewers. Any product that may be evaluated in this article, or claim that may be made by its manufacturer, is not guaranteed or endorsed by the publisher.

